# Increased levels of soluble forms of E-selectin and ICAM-1 adhesion molecules during human leptospirosis

**DOI:** 10.1371/journal.pone.0180474

**Published:** 2017-07-07

**Authors:** Loic Raffray, Claude Giry, Yoga Thirapathi, Anne-Hélène Reboux, Marie-Christine Jaffar-Bandjee, Philippe Gasque

**Affiliations:** 1 Université de La Réunion, CNRS 9192, INSERM U1187, IRD 249, CHU de La Réunion, UMR PIMIT Unité Mixte Processus Infectieux en Milieu Insulaire Tropical, Plateforme Technologique CYROI, Sainte-Clotilde, La Réunion, France; 2 Laboratoire de Biologie, CHU La Réunion site Félix Guyon, St Denis, La Réunion, France; 3 Internal Medicine Unit, CHU La Réunion site Félix Guyon, St Denis, La Réunion, France; 4 Microbiology/Virology Laboratory, CHU La Réunion site Félix Guyon, St Denis, La Réunion, France; 5 Internal Medicine Unit, GHER Hospital, St Benoit, La Réunion, France; 6 Nephrology Dialysis and Transplantation Unit, CHU La Réunion site Félix Guyon, St Denis, La Réunion, France; Cornell University, UNITED STATES

## Abstract

Leptospirosis is a multisystemic zoonotic disease with infiltration of visceral organs by *Leptospira*. The capacity of the vascular endothelium to grant immune cell recruitment and activation in target organs during the disease course remains poorly characterized. We ascertained the levels of expression of several soluble cell adhesion molecules (CAM) notably expressed by endothelial cells in human leptospirosis. We prospectively enrolled 20 hospitalized patients and compared them to 10 healthy controls. Disease severity was defined by one or more organ failures, or death. Plasmatic concentrations of soluble CAM were assessed by multiplex bead assay at the time of patient presentation (M0) and 1 month after hospital discharge. The levels of soluble E-selectin (sCD62E) and soluble intercellular adhesion molecule 1 (sICAM-1, sCD53) were significantly increased in patients compared to controls (p<0.0001) and at 1 month (p<0.0001) with median values at 978 ng/ml (interquartile ranges 787–1164; sCD62E) and 1021 ng/ml (690–1428; sCD53). At M0, Soluble P-selectin level (sCD62P) was found to be decreased with levels at 60 ng/ml (0–631) versus 711 ng/ml (343–1113) for healthy controls (p<0.05). Levels of sICAM-3 (sCD50), sVCAM-1 (vascular cell adhesion molecule, sCD106) and sPECAM-1 (platelet endothelial cell adhesion molecule, sCD31) were not different from healthy subjects at M0. This study shows that two adhesion molecules, shed as soluble forms, are elevated during the acute phase of leptospirosis: E-selectin and s-ICAM1. These molecules may interfere with the process of immune cell recruitment to clear *Leptospira* at tissue levels.

## Introduction

Leptospirosis is a zoonotic disease caused by *Leptospira* species, with a recent estimate of 1 million cases per year [1]. The course of the disease includes a broad spectrum of manifestations, from influenza-like illness to multi-organ failure with icteric hepatitis, deep thrombocytopenia, acute renal failure and more rarely intra-alveolar hemorrhage [2]. The mortality ranges from 5% to 15% especially during lung hemorrhage [1,2]. Despite increasing evidence regarding the pathological mechanisms of the disease, much remains to be demonstrated to have a better understanding of this multisystemic disease, and specially the host-pathogen interactions [3].

During the first days of the disease course, pathogenic bacteria reach the blood and evade the host immune system to disseminate to target organs [4,5]. The vascular endothelium is thus a key-actor during the bacteriemic phase of leptospirosis given its role to allow the recruitment of immune cells in tissues. Widespread endothelial damage may be a possible contributing factor to explain organ injuries, notably at the level of the lungs [6]. Endothelial cell layer disruption has been demonstrated in endothelial cell culture models [7] and in response to adhesion of the spirochete to endothelial monolayer cells [8]. Other *in vitro* studies reported a direct effect of live *Leptospira*, leptospiral components such as peptidoglycan, or recombinant proteins on the activation status of endothelial cells leading to increased expression of cell adhesion molecules (CAM) [9–12]. This was mainly demonstrated for selectins, intercellular and vascular CAMs (ICAM and VCAM respectively). Moreover, histopathological studies of human leptospirosis fatal cases showed an increased expression of ICAM and VCAM on lung endothelial cells compared to healthy controls and other non-infectious hemorrhagic cases [13].

During a canonical inflammatory response, many of the adhesion molecules expressed by endothelial cells to grant immune cell recruitment are shed from cell surfaces and accumulate as circulating soluble isoforms in plasma [14]. The expression of soluble adhesion molecules has been extensively analyzed in the setting of sepsis and septic shock as biological prognosis markers [14] but little is known in the context of human leptospirosis and as discussed later [15–17]. We were interested to evaluate the levels of several soluble adhesion molecules during the course of hospitalized cases of leptospirosis. We describe herein a prospective cohort study of human leptospiral infections tested for the levels of soluble sE-selectin, sP-selectin, sICAM-1, sICAM3, sPECAM-1, sVCAM-1 during the acute (M0) and convalescent (M1) phases of the disease.

## Material and methods

### Cohort study and ethics

Our study was conducted in a medical center of the University Hospital of Reunion Island: 10 healthy subjects and 20 patients undergoing PCR-confirmed leptospirosis were enrolled. Patients were hospitalized either in the intensive care unit or conventional medical units. They were treated according to the standards of care. Clinical and laboratory data were recorded until the point of discharge or death.

Definitions of the disease hallmarks:

- Severe leptospirosis was defined as a disease associated with death or severe organ injury. In the latter case it corresponded to patients fulfilling at least one of the following criteria during disease course: either jaundice (bilirubin > 50 μmol/L); aspartate aminotransferase (AST) increase (> 3 fold the upper normal limit); acute renal failure defined according to RIFLE “Failure” definition [18] or requirement of hemodialysis; mechanical ventilation or oxygen requirement; hypotension requiring fluid resuscitation. Patients not fulfilling these criteria were defined as non-severe leptospirosis cases.
- Leptospirosis stages: patients were enrolled during the first days after onset of symptoms and were defined as acute phase or M0 group (month 0). Biological and immunological evaluations were also performed 1 month later after patient’s discharge and defined as convalescent phase or M1 group (month 1).
- Hemorrhage was defined by presence of hemoptysis, intra-alveolar hemorrhage, purpura or other clinical bleeding: hematuria, epistaxis, rectorragia.
- Oxygen requirement was defined as use of oxygen to maintain PaO2 above 600 mmHg or SpO2 above 92%.
- Septic shock was defined according to international recognized criteria of ACPP/SCCM Consensus Conference Committee [19].
- Severe thrombocytopenia was defined as platelets inferior to 50 G/l threshold according to previous study in leptospirosis [20].

Workers at the hospital served as healthy controls and were matched with patients for age and sex.

This study was conducted according to the principles expressed in the Declaration of Helsinki and was approved by the local human ethic committee of “CHU de La Réunion” (R15018). All patients provided written informed consent for the collection of samples and subsequent analyses, performed anonymously.

### Real-time quantitative PCR analyses for diagnosis and quantification of leptospirosis

Biological specimens for diagnosis of leptospirosis were sampled at admittance of patients. Leptospires in plasma or urine were detected by quantitative real-time PCR (qPCR) using the Light cycler LC480^®^ system (Roche), and TaqMan^®^ Universal PCR Mastermix with primers and probe specific for 23S rRNA gene of *Leptospira* as detailed before [21]. For quantification of bacterial burden in plasma by PCR, serial dilutions of genomic DNA extracts from *L*. *interrogans* serogroup Icterohaemorragiae serovar Copenhageni were performed. These dilutions corresponded to concentrations from 4 x 10^6^ to 4 bacteria/ml and the number of bacteria per ml in plasma samples was inferred from the cycle threshold (Ct) values of PCR according to the log-transformed standard curve, as detailed in previous reports [22].

### Multiplex soluble adhesion molecule measurements

The quantitative evaluation of relevant soluble adhesion molecules was performed with the commercial bead based assay FlowCytomix Human Adhesion 6plex kit (ref. BMS812FF, Bender MedSystems^®^) on blood sampled at admission. Procedure was performed according to manufacturer’s indications and enabled to measure the concentrations of 6 soluble factors: sE-selectin, sP-selectin, sICAM-1, sICAM-3, sPECAM-1, sVCAM-1. Briefly, plasma was incubated with beads coated with specific antibodies toward the soluble factors. After washing to eliminate free beads, phycoerythrin-conjugated antibodies specific for the soluble factors were incubated with the mixture. After washing, the beads are distinguished with a flow cytometer according to their size and specific autofluorescence while the mean fluorescence intensity (MFI) of PE-antibodies linked to soluble factors is determined. The concentrations of each soluble factor were inferred from MFI, according to standard curves. Cytometry analysis was performed with the Becton Dickinson C6 accury^™^ flow cytometer, and data extracted from BD Accury^™^ C6 software version 1.0.

### Statistics

Data are expressed as medians and interquartile ranges for quantitative variables; and as numbers and percentages for qualitative variables. Owing to non-Gaussian distribution, statistical significance of difference between groups was determined by non-parametric Mann-Whitney U-test for continuous variables and by Khi-2 square test for qualitative variables. For paired data the Wilcoxon non-parametrical test was used. The Spearman test was used to analyze correlations among variables. *P*-values below 0.05 were considered statistically significant. Statistics were performed with GraphPad Prism^™^.

## Results

### Cohort and patients’ clinical and biological data

The clinico-biological data of leptospirosis patients and age- and sex-matched healthy controls are indicated in Table 1. Patients exhibited classical hallmarks of acute leptospirosis with moderate to severe organ injuries. From the time of reported first symptoms the median delays to hospitalization and antibiotic treatment were 5 days (interquartile 4–5). Among the infected patients, 14 were considered as severe leptospirosis cases according to the definition of an infectious disease with organ failure as indicated above. These severe cases corresponded to: 11 cases of severe acute renal failure among which 9 required renal replacement therapy during a median of 3 days (2.8–6); 7 cases had hemodynamic failure among which 4 required vasopressor drugs; 6 cases required oxygen administration and 3 had mechanical ventilation. One patient deceased with multi-organ failure and as a consequence of septic shock within 2 days of medical care. When comparing the groups of severe (14) and non-severe (6) leptospirosis patients we found additional significant differences for surrogate markers that were not used initially in the definition of severe cases: thrombocytopenia (p = <0.001), neutrophilia (p = 0.01) and creatinine phosphokinase (CPK) levels (p = 0.009) which were significantly more pronounced in the severe cases compared to non-severe forms of leptospirosis. Bleeding was also significantly associated to low platelet counts (p = 0.02). Plasma bacterial load inferred from PCR Ct values was higher in the severe group although not reaching statistical significance: median of 7.8x10^2^/ml compared to 5.2x10^1^/ml (p = 0.054). The length of hospital stay was also longer for severe cases: 7 days in median instead of 4 days (p = 0.005). Noteworthy, the time to initiate the antibiotic treatment was not significantly different between the two groups with 5 days in median for severe and non-severe leptospirosis groups.

**Table 1:** Characteristics of leptospirosis patient group at admittance and comparison to healthy subjects.

characteristic (units)	Controls	Leptospirosis cases	control vs. leptospirosis
Number of individuals	10	20	
Ratio M/F	9/1	19/1	NS
Age (years)	43.4 (29.3–53.6)	44.3 (27.5–55.8)	NS
Neutrophils (10^9^/L)	3.6 (3.2–3.9)	9 (7.3–10.3)	**<0.0001**
Lymphocytes (10^9^/L)	2.2 (1.8–2.7)	0.6 (0.5–0.9)	**<0.0001**
Monocytes (10^9^/L)	0.5 (0.4–0.6)	0.6 (0.4–0.8)	NS
Platelets (10^9^/L)	233 (214–253)	53 (37–102)	**<0.0001**
Creatinine (μmol/L)	83 (20–89)	152 (103–394)	**<0.0001**
Total bilirubin (μmol/L)	9 (7–11)	41 (27–123)	**<0.0001**
AST (IU/L)	25 (22–31)	89 (52–169)	**0.0002**
CPK (IU/L)	140 (115–206)	1410 (910–4365)	**0.0006**
CRP (mg/L)	1 (0.4–2)	212 (187–304)	**<0.0001**
Plasmatic bacterial load* (bact./ml)	NA	344 (61–2566)	

Data are expressed as medians (interquartile ranges). Statistics between 2 groups were performed with nonparametric unpaired tests (Mann-Whitney U-test) for quantitative variables, and with Khi-2 square test for categorical data. P value inferior to 0.05 was considered significant.

*Plasmatic bacterial load was inferred from plasmatic PCR Ct values according to the log-transformed standard curve.

(AST = aspartate aminotransferase; CPK = creatine phosphokinase; CRP = C-reactive protein; NS = not significant)

**Fig 1:**
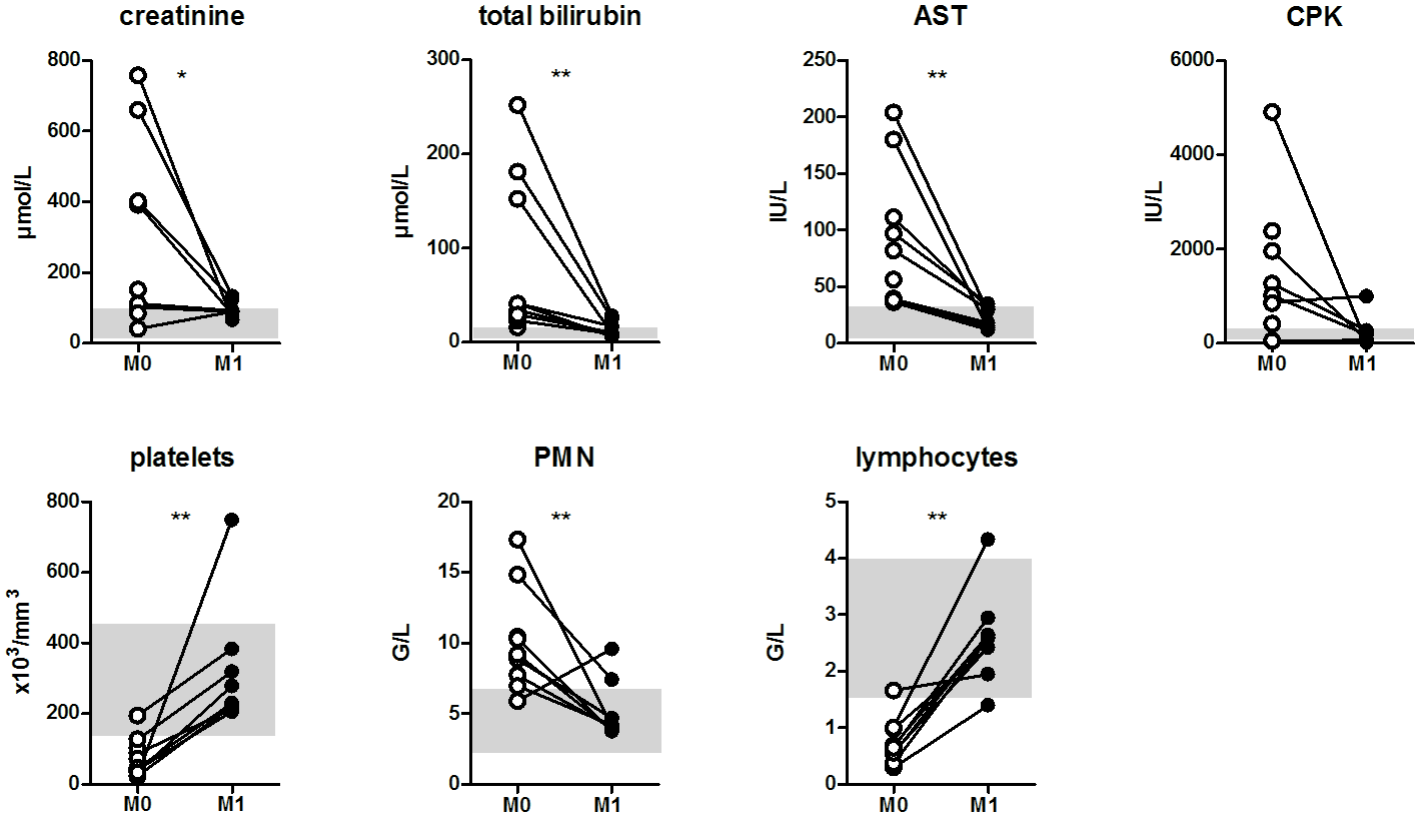
Correction of blood cell counts and tissue-injury biomarkers at 1 month post-leptospirosis. Evolution of blood cell counts and surrogate biomarkers of tissue injury between acute (M0, empty circles) and convalescent phase, 1 month later (M1, black circles) for 10 patients. Gray areas indicate normal values ranges. Two values for M1 patient CPK (creatine phosphokinase) measurement could not be included. Comparisons with non-parametric Wilcoxon paired test. * and ** indicate P-value inferior to 0.05 and 0.01 respectively.

Among the 20 patients, 10 patients agreed to be evaluated 1 month after hospital discharge, of whom 7 were considered as severe cases at M0. The assessment of M1 outpatient group indicated a rapid correction of the symptoms and biological alterations were not significantly different from those of the control group (Fig 1).

### Shedding of adhesion molecules into soluble forms during leptospirosis

**Fig 2:**
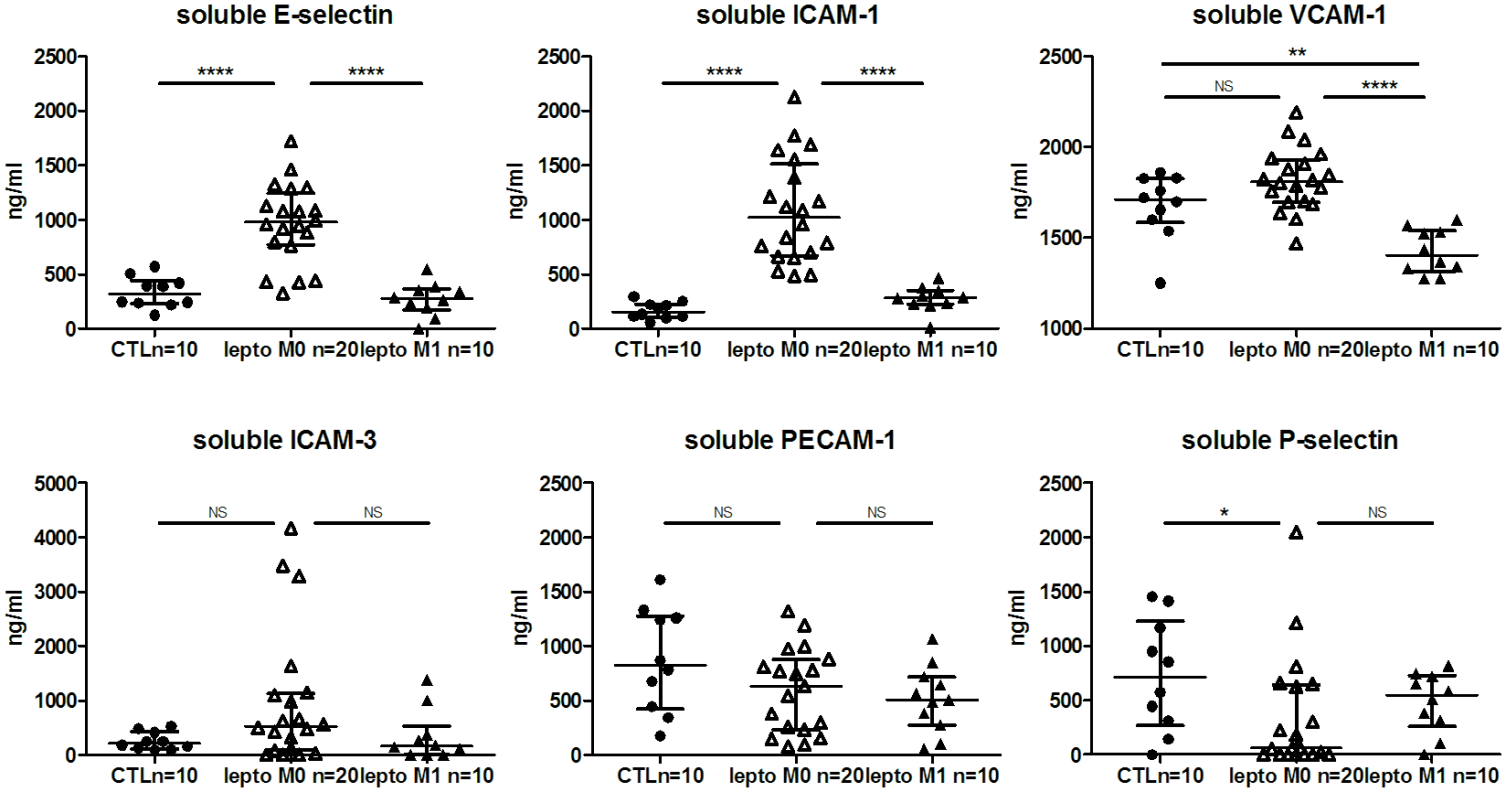
Levels of soluble E-selectin and soluble ICAM-1 are increased during the acute phase of human leptospirosis. Levels of soluble (shed) adhesion molecules are determined in patients and controls using multiplex microbeads array. Circles represent controls (n = 10), empty triangles: patients during acute phase (n = 20) and black triangles: patients at M1 convalescent phase (n = 10). The largest horizontal bars indicate the median value, upper and lower bars the interquartile ranges. Comparisons with non-parametric Mann-Whitney test. *, **, **** indicate P-value inferior to 0.05, 0.01 and 0.0001 respectively.

The levels of soluble adhesion factors implicated in leukocyte recruitment are indicated in Fig 2. We found a clear increase for sE-selectin and sICAM-1 levels when comparing leptospirosis patients at M0 and healthy subjects (controls) (p<0.0001). The levels of soluble E-selectin were 978 ng/ml in median for patients (interquartile ranges 787–1164) and 314 ng/ml (236–412) for healthy controls. sICAM-1 levels were also elevated with median values of 1021 ng/ml (690–1428) for patients and 155 ng/ml (111–216) for controls. These elevated values returned to the levels of the control group for the evaluation at M1 after discharge. Concerning sVCAM-1, there was a trend to higher levels in patients with leptospirosis, although not of statistical significance; whereas the values at M1 were lower when compared to controls and M0 patient group. sP-selectin was the only tested molecule with lower expression levels during the acute phase of the disease compared to controls: 60 ng/ml (0–631) versus 711 ng/ml (343–1113) (p<0.05).

Comparison between the severe leptospirosis cases (n = 14) and non severe cases (6) showed no difference for the levels of each of the adhesion molecules soluble forms tested. Of critical note, the severity of the disease was established from a clinical standpoint (i.e. organ failure as aforementioned). Moreover, the elevated levels of the adhesion molecules were not associated with hospitalization in intensive care units or hospitalization length.

**Fig 3:**
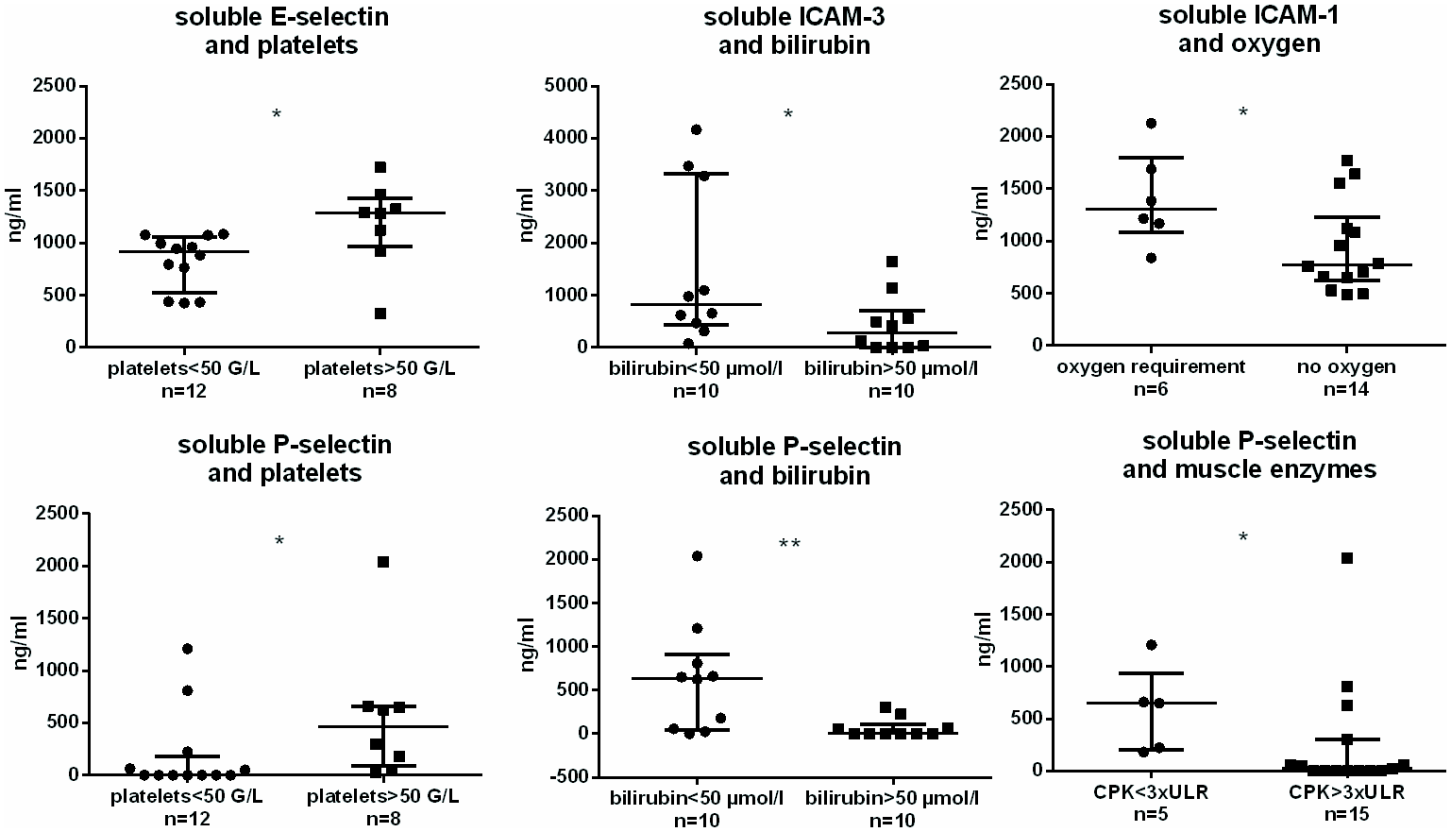
Distinct associations between shed adhesion molecules and organ injuries in 20 leptospirosis patients. Levels of soluble (shed) adhesion molecules are determined among leptospirosis M0 group (n = 20) using multiplex microbeads array. The largest horizontal bars indicate the median value, upper and lower bars the interquartile ranges. Comparisons with non-parametric Mann-Whitney test. * and ** indicate P-value inferior to 0.05 and 0.01 respectively. CPK = creatine phosphokinase; ULR = upper limit range.

We next further analyzed whether the levels of sCAMs were possibly associated to the different organs, or surrogate markers, affected in leptospirosis (Fig 3 and S1 Table). Counterintuitively, we found that patients in the group with very low platelet counts (<50 G/L) had low levels of sE-selectin when compared to patients with higher platelet counts (>50 G/L) (p = 0.02) (Fig 3). This association was also observed for sP-selectin levels and platelet counts. Thus, we found a positive correlation between platelet counts and sP-selectin levels: p = 0.02 and r = +0.25. When we used bilirubin as another biomarker of disease severity we found a significant association between low levels of sICAM-3, sP-selectin and high levels of bilirubin (>50 μmol/L) (Fig 3). A significant association was found also between sICAM-1 and oxygen requirement as well as between levels of sP-selectin and CPK. The amount of circulating bacteria (assessed by PCR) was inversely correlated to sICAM-3 values (p = 0.014, r = -0.47) and not correlated to other soluble adhesion molecules.

## Discussion

The aim of this study was to assess indirectly the relative contribution of vascular endothelial cells in human leptospirosis by measuring the levels of several soluble CAMs in blood. We analyzed plasma levels of several of the key CAMs shed as soluble forms. These sCAMs have been considered prognostic biomarkers of severity of systemic inflammation but the clinical importance of monitoring such changes remains to be established [23]. We herein report a major increase in sE-selectin and sICAM-1 levels in patients with leptospirosis, whereas sP-selectin levels were lower compared to control healthy subjects. Our results indicate that endothelial cells may be activated during leptospirosis and that yet ill-characterized mechanisms are likely to contribute to the release of soluble forms of the CAMs through the activity of sheddases, membrane-bound proteases or caspases.

E-selectin is the most specific CAM expressed by endothelial cells, together with ICAM-1 and VCAM-1. E-selectin belongs to the selectin family of CAM and mediates the first step of leukocytes (PMN) and platelets rolling onto the endothelium through its ligands P-selectin glycoprotein ligand 1 (PSGL1) mainly and E-selectin ligand (ESL1) [24,25]. Expression of E-selectin is upregulated upon inflammatory conditions and this cell surface molecule can be cleaved through an active process of shedding mediated by caspases and giving rise to soluble isoforms. It is recognized that sE-selectin level reflects the activation status of endothelial cells [26]. Interestingly, sE-selectin in sera has been shown to retain the ability to adhere to its ligand on circulating leukocytes, thus decreasing the ability of immune cells to be mobilized at the site of inflammation. This decoy mechanism is thought to constitute a self-limiting process to avoid detrimental inflammation due to uncontrolled leukocyte recruitment in tissues. We believe that this paradigm proposed in many infectious diseases could also be involved as a mechanism to control leukocyte recruitment in leptospirosis.

Shedding has also been described for the other CAM studied here [14]. ICAM-1 and VCAM-1 are members of the immunoglobulin superfamily and bind to integrins LFA-1 (CD11a/CD18), CR3 (CD11b/CD18), and VLA-4 during the firm adhesion and transendothelial migration steps. Expression of these CAMs is constitutive and also inducible after the action of inflammatory cytokines (TNFα, IL-1, and IFNγ) or LPS exposure [14].

*In vivo* studies addressing the mechanisms of endothelial cell activation/damage during leptospirosis are scarce but have already yielded insightful information. In a study comparing « typhus-like » infectious diseases in Thai patients, the authors found elevation of sE-selectin, sICAM-1 and sVCAM-1 levels in 24 leptospirosis cases compared to healthy subject controls. Leptospirosis patients had high levels of sE-selectin and sICAM-1, but these parameters were not assessed as prognosis markers for leptospirosis [16]. However, in Liborio et al.’s study, the significant elevation of sICAM-1 and glycocalix in leptospirosis patients was correlated to renal damage and possibly as a consequence of endothelial damage rather than the activation status according to the authors [15]. Moreover, Goeijenbier et al. observed that Von Willebrand factor and soluble E-selectin plasma levels were elevated within the 14 days period following initial symptoms of leptospirosis in humans [17]. Soluble E-selectin levels were correlated to mortality, even though not linked to bleeding. Surprisingly, these authors experienced *in vitro* that *Leptospira*-treated human umbilical vein endothelial cells (HUVECs) did not express increased levels of sE-selectin and sICAM-1, although data were not shown. *In vivo* and in the context of a vascular inflammation, the process of shedding may likely be mobilized by activated immune cells to cleave the CAMs expressed by endothelial cells. Sheddases are cell-derived enzymes that cleave extracellular portions of the endothelial CAMs. The expression and plausible roles of specific sheddases in leptospirosis are unknown and experiments along these lines are now highly warranted.

Our results largely corroborate previously published data [15–17]. We demonstrated that sE-selectin and sICAM-1 levels were increased during non-severe and severe forms of human leptospirosis. We did not find a correlation between the levels of sE-selectin and global clinical disease severity (multiple organ failure or death) in contrast to the data reported in the Indonesian’s cohort [17]. Of note, the Indonesian’s study included 14/52 fatal cases compared to one death in our cohort and they did not assess other severity factors and therefore comparison between both studies is not possible. Of note, high sE-selectin levels have also been linked to severe outcome in other sepsis studies [14,27]. A new element described in our study is the normalization of sE-selectin levels within 1 month after initial assessment. At that time patients had probably recovered from the main tissue injuries as well as from the systemic inflammatory response. Therefore, our patients can be considered as their own control to demonstrate that the process of shedding to modulate the expression and possibly the function of endothelial CAM is contemporary to leptospiral infectious process.

The increased level of sICAM-1 is consistent with previous results of the Thai patients [16] and of a study performed on 46 patients during a leptospirosis outbreak in Brazil [15]. Our results indicated values 3 times superior to the two previous studies. These studies reported a 2-fold increase over control and compared to a 6-fold increase in our work. The discrepancies are possibly depending on several factors such as differences in measurement techniques, the disease severity or the bacterial strain. Liborio et al. demonstrated a positive and independent association between sICAM-1 and acute kidney injury defined by KDIGO criteria. We did not observe this association when we used renal replacement therapy and the definition of acute renal failure according to RIFLE criteria and which are commonly used to define critical kidney illnesses [18]. The only association that we found for sICAM-1 in our cohort was the need for oxygen. This could be linked to the activation status of pulmonary endothelial cells as observed in the autopsy analysis carried out by Del Carlo Bernardi and colleagues [13].

To our knowledge, soluble P-selectin levels had never been explored in leptospirosis prior to our study. Counterintuitively, levels of this CAM were decreased. Moreover several patients had below detectable levels of sP-selectin in their plasma. sP-selectin is usually up-regulated in plasma of septic patients [14,28]. The degree to which sP-selectin in plasma is derived from platelets or endothelial cells is difficult to address. Indeed, other cells of the vascular bed could release soluble CAMs: platelets are abundant sources of membrane and soluble forms of P-selectin as well as PECAM-1. Leukocytes (monocytes, neutrophils, lymphocytes) are known to express high levels of ICAM-3 and to a lesser extent PECAM-1. Hence, the interpretation of our results may be more complex taking into account the contribution of several cell types releasing soluble forms of the CAM. Interestingly, we found a correlation between the levels of sP-selectin and platelets indicating a plausible role for shedding of P-selectin from platelets. Moreover low sP-selectins levels were associated with high bilirubinemia and muscle enzymes (CPK) increases. As major thrombocytopenia is a hallmark of severe acute leptospirosis [29,30], the low levels of sP-selectin observed here are possibly a consequence of platelet decrease. Data from Hantavirus infection, a viral hemorrhagic fever with profound thrombocytopenia, revealed a positive correlation between platelet cell counts and sP-selectin levels, paralleling our results [31].

As stated above, the measurement of the levels of soluble CAMs does not allow distinguishing whether this is due to either endothelial cell activation or other causes such as cell damage or cell death. Moreover, these sCAMs may also be derived from activated platelets. In leptospirosis, we could also speculate that active shedding may be due to leptospiral proteases as described [32,33]. Studies of other endothelial activation markers in the setting of leptospirosis, such as angiopoietin and dimethylarginin, should be important to address the link with pathogenesis [34].

In conclusion, our study provides new data regarding the monitoring of six major soluble adhesion molecules that may be important to explain the pathophysiological mechanisms of leptospirosis. We did not find a strong correlation between the levels of these soluble CAMs and the canonical cellular and molecular biomarkers of clinical disease severity. The main changes in molecule levels were observed for CAMs specific of endothelial cells such as soluble E-selectin and ICAM-1. If the changes are not specifically associated to global clinical disease severity, they clearly indicate an important role of the endothelium during the infectious process. CAMs derived from platelets and the capacity of *Leptospira* to control platelet’s functions may also be involved in pathogenesis of leptospirosis and should be addressed in further studies.

## Supporting information

S1 Table Comparison between tissue injury groups for levels of soluble adhesion molecules in 20 leptospirosis patients. Comparisons with non-parametric Mann-Whitney test between the group with and without the indicated organ injury. ULR = upper limit range; other definitions: see Methods section.
(DOCX)
