# Supplementary material for: Increased levels of soluble forms of E-selectin and ICAM-1 adhesion molecules during human leptospirosis
Source: PLoS One. 2017 Jul 7;12(7):e0180474. doi: 10.1371/journal.pone.0180474 (PMC5501535; doi:10.1371/journal.pone.0180474)
Supplement: S1 Table — Comparisons with non-parametric Mann-Whitney test between the group with and without the indicated organ injury. ULR = upper limit range; other definitions: see Methods section. (DOCX) [file pone.0180474.s001.docx]

**S1 table**. **Comparison between tissue injury groups for levels of soluble adhesion molecules in 20 leptospirosis patients**

|  | **sE-selectin** | **sICAM-1** | **sICAM-3** | **sPCAM-1** | **sP-selectin** | **sVCAM-1** |
| --- | --- | --- | --- | --- | --- | --- |
| **Hemorrhage (n=5)** | NS | NS | 0.06 | NS | NS | NS |
| **Platelets<50 G/l (n=12)** | **0.02** | NS | NS | NS | **0.03** | NS |
| **Bilirubinemia>50 µmol/l (n=10)** | 0.07 | NS | **0.04** | NS | **0.01** | NS |
| **Liver enzymes >3xULR (n=10)** | NS | NS | NS | NS | NS | NS |
| **Muscle enzymes >3xULR (n=15)** | NS | NS | NS | NS | **0.03** | NS |
| **Acute renal failure according to RIFLE (n=11)** | NS | NS | NS | NS | NS | NS |
| **Renal replacement therapy (n=9)** | NS | NS | NS | NS | NS | NS |
| **Oxygen requirement (n=6)** | NS | **0.03** | NS | NS | NS | NS |
| **Septic shock (n=5)** | NS | NS | 0.08 | NS | NS | NS |

Caption: comparisons with non-parametric Mann-Whitney test between the group with and without the indicated organ injury. ULR= upper limit range; other definitions: see methods section.
